# Alterations of PAC-based resting state networks in Parkinson’s disease are partially alleviated by levodopa medication

**DOI:** 10.3389/fnsys.2023.1219334

**Published:** 2023-08-01

**Authors:** Sean Mertiens, Matthias Sure, Alfons Schnitzler, Esther Florin

**Affiliations:** ^1^Institute of Clinical Neuroscience and Medical Psychology, Medical Faculty, Heinrich Heine University Düsseldorf, Düsseldorf, Germany; ^2^Department of Neurology, Center for Movement Disorders and Neuromodulation, Medical Faculty, Heinrich Heine University Düsseldorf, Düsseldorf, Germany

**Keywords:** Parkinson’s disease, resting state networks, magnetoencephalography, phase-amplitude coupling, levodopa

## Abstract

**Introduction:**

Parkinson’s disease (PD) is a neurodegenerative disorder affecting the whole brain, leading to several motor and non-motor symptoms. In the past, it has been shown that PD alters resting state networks (RSN) in the brain. These networks are usually derived from fMRI BOLD signals. This study investigated RSN changes in PD patients based on maximum phase-amplitude coupling (PAC) throughout the cortex. We also tested the hypothesis that levodopa medication shifts network activity back toward a healthy state.

**Methods:**

We recorded 23 PD patients and 24 healthy age-matched participants for 30 min at rest with magnetoencephalography (MEG). PD patients were measured once in the dopaminergic medication ON and once in the medication OFF state. A T1-MRI brain scan was acquired from each participant for source reconstruction. After correcting the data for artifacts and performing source reconstruction using a linearly constrained minimum variance beamformer, we extracted visual, sensorimotor (SMN), and frontal RSNs based on PAC.

**Results:**

We found significant changes in all networks between healthy participants and PD patients in the medication OFF state. Levodopa had a significant effect on the SMN but not on the other networks. There was no significant change in the optimal PAC coupling frequencies between healthy participants and PD patients.

**Discussion:**

Our results suggest that RSNs, based on PAC in different parts of the cortex, are altered in PD patients. Furthermore, levodopa significantly affects the SMN, reflecting the clinical alleviation of motor symptoms and leading to a network normalization compared to healthy controls.

## 1. Introduction

Parkinson’s disease (PD) leads to degeneration and functional impairment throughout the brain, causing both motor and non-motor symptoms (Lang and Lozano, 1998). This widespread dysfunction also affects different cortical networks that make up short and long-range communication throughout the brain (Cerasa et al., 2016). The key neuropathological characteristic of PD is the decline of dopaminergic neurons (Mullin and Schapira, 2015), and the resulting dopamine deficiency in basal ganglia could be linked to weaker connectivity within resting state networks (RSN) such as the Default Mode Network (DMN) (Tahmasian et al., 2017). A single measurement can be used to obtain RSN, and various functional networks can be determined, including motor, vision, emotional control, and attention (Yeo et al., 2011). Since the neural impairments and clinical representation in PD also involve different functional systems, analyses of RSN are suitable to determine PD-relevant changes.

In the literature, fMRI imaging is seen as the “gold standard” for identifying these RSNs in individuals (Biswal et al., 1995). For some time, data-driven approaches for RSN detection have been proposed for magnetoencephalography (MEG) and electroencephalography (EEG) data, showing a robust correlation with fMRI networks (Brookes et al., 2011; Florin and Baillet, 2015). In addition, Schneider et al. (2020) recently found that RSNs derived from scalp-EEG using a combination of amplitude envelope and independent component analysis (Envelope-ICA) (Brookes et al., 2011) are altered in PD patients and sensitive to levodopa medication (Schneider et al., 2020).

Here we investigate these network alterations further using instead of Envelope-ICA, an approach based on phase-amplitude coupling (PAC) (Florin and Baillet, 2015). With MEG, we reach a higher spatial resolution than with EEG, while deriving cortical networks from PAC produces networks more closely resembling those from fMRI (Pelzer et al., 2021). Furthermore, Swann et al. (2015) and De Hemptinne et al. (2013) showed that PAC is elevated in PD patients without medication compared to both healthy controls (HC) and PD patients with medication over the sensorimotor cortex. In addition, the decrease in PAC after levodopa administration is correlated with the improvement of bradykinesia-related subitems in the Unified Parkinson’s Disease Rating Scale motor (MDS-UPDRS-III) score (Miller et al., 2019). PAC was, therefore, recently proposed as a possible biomarker of the parkinsonian state (Miller et al., 2019), establishing a link between neuroscientific research and clinical application. In PD, PAC is modulated, especially within beta, as the low-frequency component of the PAC (De Hemptinne et al., 2013; Swann et al., 2015). However, these beta oscillations have a non-sinusoidal shape, which interferes with PAC estimation (Cole and Voytek, 2017).

In this study, we compared well-matched groups of PD patients with HC. PD patients were measured with and without medication to investigate whether PAC-RSNs are altered in PD and whether levodopa has a normalizing effect on these networks, as levodopa leads to symptom relief in PD. We also hypothesize that differences will be evident in different functional RSN, as PD patients have both motor and non-motor symptoms (Przedborski, 2017). We quantified the changes in cortical activation using jackknife statistics.

## 2. Materials and methods

### 2.1. Participants

We recorded 28 PD patients. Inclusion criteria were clinically confirmed PD with positive motor response to levodopa or apomorphine and age ranging between 40 and 70 years, and exclusion criteria were any other severe neurological or psychiatric disease, a Parkinson-plus-syndrome as well as severe frontal executive dysfunction and hypokinetic-rigid movement disorders. Patients had been diagnosed with PD and were selected for DBS treatment within the nucleus subthalamicus according to the guidelines of the German Society for Neurology. Furthermore, patients were not selectively chosen for a phenotype, but patients with severe head tremor were excluded because it affects the quality of measurement. From the originally 28 patients, five patients were excluded because medication OFF and/or ON data quality was not sufficient for further analysis due to motion artifacts or the recordings yielded less than 10 min of usable MEG data per condition. We used the MDS-UPDRS-III motor score to assess motor dysfunction (Goetz et al., 2008) and levodopa response based on the change in the MDS-UPDRS-III motor score. The MDS-UPDRS-III score was obtained on the day of the MEG-measurement.

For the HC group, we recruited 25 participants in the age between 40 and 70 years, which were not regularly taking medication influencing the central nervous system. One participant could not be included in the study due to excessive motion artifacts.

All participants completed a Beck Depression Inventory (BDI-II) test to screen for depression and the mini-mental status test (MMSE) (Folstein et al., 1975) to assess cognitive abilities. Of the HC, 23 were right-handed (1 left). In the PD group, 22 subjects were right-handed (1 both) according to the Edinburgh Handedness Inventory (EHI) (Oldfield, 1971). For detailed descriptive statistics, see Table 1.

**TABLE 1:** Descriptive statistics.

	Control (*n* = 24)	PD (*n* = 23)
Age (years)	63.2 ± 5.1	60.2 ± 7.9
Gender	Female: 8 Male: 16	Female: 7 Male: 16
MMSE (points)	29.6 ± 0.6	28.7 ± 1.6
BDI-II (points)	4.3 ± 4.1	11.7 ± 7.9
EHI score (points)	73.5 ± 32.5	75.4 ± 25.1
Levodopa dosage (mg)	–	169.0 ± 37.0
Disease duration (years)	–	7.0 ± 3.9
MDS-UPDRS-III score OFF (points)	–	36.0 ± 11.0
MDS-UPDRS-III score ON (points)	–	21.6 ± 9.6
MDS-UPDRS-III improvement (off-on) (points) (percentage)	–	14.4 ± 8.4 40.8 ± 18.9
Number of PD patients with dopamine agonists	–	9
L-dopa equivalent dose (LEDD) for dopamine agonists (mg)	–	62.7 ± 112.9

Descriptive statistics of study groups. Values are referring to the mean ± standard deviation.

All participants gave written consent to participate in the study. The study was approved by the Local Ethics Committee (study no. 5608R) and conducted in accordance with the Declaration of Helsinki.

### 2.2. MEG recording

Participants were seated in a magnetically shielded room. MEG measurements were done with a 306-channel Elekta system (Elekta Vectorview, Elekta Neuromag, Finland) at the University Clinic in Düsseldorf.

In addition to the MEG signals, we recorded electrocardiography, electrooculography, and pupil diameter (iView X 2.2, SensoMotoric Instruments, Teltow, Germany) in order to identify and correct artifacts during data preprocessing.

At the beginning of every MEG session, four head position indicator coils were placed on the patient’s head, and their position was digitized using the Polhemus system (Polhemus Isotrack, Colchester, CT, USA). In addition, the fiducial points (nasion, left preauricular, and right preauricular) and the participant’s head surface were digitized using the same system to allow for better registration of the head position within the MEG and the participant’s MRI. Before each MEG recording block, the participant’s head position inside the MEG helmet was registered using the four coils.

For each participant, three blocks of 10 min of eyes-open resting state data per condition were recorded with a sampling rate of 2,400 Hz, a lowpass filter of 800 Hz, and DC correction. Recordings were performed in a seated position, while a cardboard fixation cross was presented to limit eye movement and visual attention bias. After each 10-min section, the participant had the opportunity to take a break to reduce potential fatigue. Additionally, 5 min of empty room data were acquired for each MEG session on the same day. To ensure an OFF medication state for the patient’s measurement, oral PD medication was discontinued at least 12 h before the start of the measurement. For the following ON block, one and a half times the morning levodopa dose was given as fast-acting soluble levodopa. To ensure a stable ON state, we waited until clinical improvement occurred. Two patients that were present in both PD groups were measured in “best medical on,” meaning the patients took their regular dopaminergic medication and were measured at the time of maximal clinical effect.

For the PD patients, the 3T T1-MRIs, which were obtained as part of the clinical routine after the MEG, were used. For HC, T1-MRIs were acquired in the following days or weeks after the MEG measurement (MAGNETOM Prisma, Siemens Healthcare, Erlangen, Germany; 3 Tesla). These T1-MRIs were used to reconstruct the cortical surface of each participant with FreeSurfer (Dale et al., 1999).

### 2.3. Data preprocessing

Data analysis was performed with the Matlab-based (PRID:SCR_001622; version R2019a; The MathWorks, Inc., Natick, MA, USA) Brainstorm software (RRID:SCR_001761; Tadel et al., 2011), which is documented and freely available for download online under the GNU general public license.^1^

All recordings have been visually checked by at least one researcher; in case of low data quality (such as excessive noise or noise that could not be removed using the methods mentioned below), the data was reviewed independently by another researcher to obtain a consensus on the usability of the data. For all recordings the signal-space projectors (SSP) provided by the Neuromag system were applied to reduce the environmental noise. Frequently occurring artifacts such as eye-blinks and cardiovascular artifacts were removed using Brainstorms built-in SSP functions (Uusitalo and Ilmoniemi, 1997). Movement and other artifacts that could not be cleaned with SSPs were removed by excluding these parts of the recording from further processing. In the case that artifacts appeared mainly in one MEG-channel, the MEG-channel in question was removed. On average, 10.1 ± 5.2% of the MEG-channels were removed per run. If there were too many artifacts in one of the individual measurement blocks that could not be removed from the data (e.g., jumps in the MEG sensor or head movement), then this block was completely excluded from further analysis.

Line noise in a range from 50 to 600 Hz with 50 Hz steps was removed using a notch filter. After artifact removal, the data were resampled to 1,000 Hz for the following steps.

### 2.4. Source reconstruction

We used the overlapping spheres head model available in Brainstorm (Huang et al., 1999). Source reconstruction was done inside Brainstorm using a linearly constrained minimum variance beamformer (Van Veen et al., 1997) on the participant’s individual cortex surface obtained from FreeSurfer at a resolution of 15,002 vertices.

### 2.5. megPAC calculation

For the extraction of RSNs, we used the method from Florin and Baillet (2015). All source data for a participant (and in the case of patients for each medication state separately) were concatenated in time. PAC between the low frequencies from 2 to 30 Hz and the high frequencies from 80 to 150 Hz was calculated for each source time series using the method from Özkurt and Schnitzler (2011). Based on this, the frequency pair with the maximal PAC value was determined and used to extract the megPAC time series, which is the amplitude of the corresponding high-frequency signal interpolated between the peaks and the troughs of the low-frequency signal [for details on obtaining the megPAC time series see Florin and Baillet (2015)]. This was done for each vertex in the individually reconstructed cortical source space and down-sampled to 10 Hz. We then used Brainstorm’s built-in methods to project these individual datasets on the ICBM152 standard brain (Fonov et al., 2011) and applied a spatial smoothing filter using Brainstorm’s processing pipeline of 7 mm full width at half maximum to the cortex surface. We then calculated the correlation between all cortical time series concatenated across participants. The cortical RSNs were then extracted using a singular value decomposition after a dimensionality reduction on the correlation matrix (see Florin and Baillet, 2015). We also performed the same analysis on simulated Gaussian noise data, and the first component was used as a projector to control the noise (see Florin and Baillet, 2015). The resulting principal modes were cortical maps with values between 0 and 1, which we will refer to as coupling strength.

### 2.6. Group network comparison

To compare the RSNs between HC, PD-ON, and PD-OFF, we used a jackknife approach (Stute, 1996; Efron and Stein, 2007). Therefore, we repeatedly calculated RSNs, each time leaving out one of the participants. That resulted in 23 jackknife runs for each PD condition and 24 runs in the HC group, respectively, with each participant missing in one of these runs (every participant was left out exactly once).

From each of the jackknife runs, we obtained nine networks. To quantify the similarity of these networks to known RSNs (“templates”), we used a phi coefficient between a list of template networks and the given component as a measure of the correlation between two binary variables (activity/no activity) (Yule, 1912). This step also helped in pre-sorting network components since networks would not necessarily be in the same order in all jackknife runs.

**FIGURE 1:**
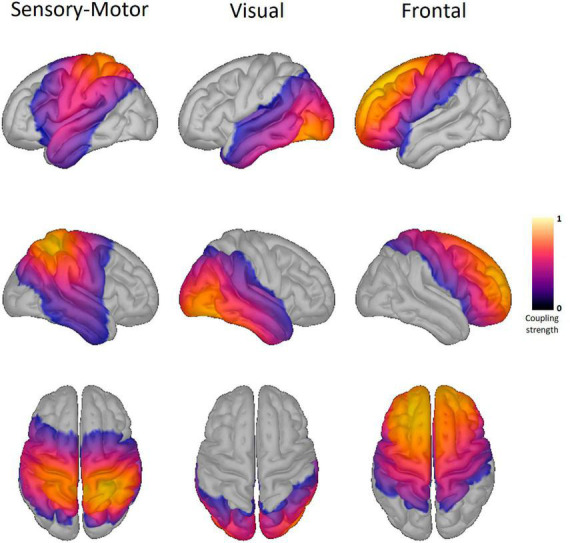
Resting-state networks of healthy controls. Shown here are the three resting-state networks (RSN) of the healthy controls. The same RSN are also detected in the two Parkinson conditions. The columns display the sensory-motor, visual, and frontal RSN. The color scale marks the coupling strength from 0 to 1, with a warmer color indicating a higher coupling strength. No threshold was applied.

As templates, we calculated RSNs over all participants in each of the three groups, resulting in nine network components for every template. Of these components, we selected three components that we could detect in all three groups (i.e., HC, PD-OFF, and PD-ON). These networks are further referred to as sensorimotor (SMN), visual, and frontal networks due to their spatial distribution. These templates were also used in the jackknife pseudo-value calculation (see Figure 1).

For the assignment of networks to the corresponding template, we processed each of the jackknife runs in the following way:

1. Threshold the coupling strength at every cortical source to a minimum of 0.4 (values are normalized to a range between 0 and 1). All values below the threshold are set to 0, and all values above to 1, resulting in a binary map of the template.
2. For the two binary maps, we calculate the phi-coefficient *R* between every network found in the jackknife run and every template network.
3. The network with maximum positive *R* was assigned to the template. If the network matched with two templates, we determined the maximum difference between the *R*-values of both matches. If the difference was at least 0.2, the match with the highest *R* was chosen. If the difference was smaller, the best match was determined by visual comparison.
4. Both the network and the template are removed from the list of available networks, so they cannot be reassigned during the current run.

The result of this assignment step is a file for every template holding all matched components for this template from every participant.

An estimate of variance was determined using the jackknife method, analogous to Sure et al. (2023). For each vertex, we used a paired two-sided *t*-test for the comparison between PD-OFF and PD-ON and an unpaired *t*-test for the comparison between HC and PD. We corrected for multiple comparisons using the false discovery rate (FDR) correction. In addition to the vertices, we also corrected for the number of networks and conditions. Results were considered significant after correction at the 0.05 level. The anatomical regions referred to in the results section are based on the Desikan-Killiany atlas as provided in Brainstorm (Desikan et al., 2006). For the functional segregation of the sensorimotor cortex, we refer to Mayka et al. (2006). We will not discuss changes in the subcortical parts of the brain since MEG sensitivity is lower in these areas in comparison to the cortex and might produce false data.

Descriptive and test statistics on participant data have been calculated using the JASP Statistics Software (JASP Team, 2023).

### 2.7. Comparison of PAC frequencies

We were also interested in the phase-driving low-frequency component of the PAC signal and whether the frequency spectrum would change between groups and conditions.

For this analysis, we determined the maximum PAC value in the frequency plane of low and high frequencies for every vertex of each participant. To determine whether this value was significantly different from zero, we generated 100 PAC values from random noise data with a 1/f characteristic (Kasdin, 1995) and data length corresponding to each participant. We accepted a PAC value from the participant data if it was above the 95th percentile of the noise data’s value. For this PAC value, we then determined the corresponding low frequency. These low frequencies were then compared between the conditions for each vertex using an independent two-sided *t*-test as provided in Brainstorm’s processing pipeline. FDR correction was used to correct for multiple comparisons. Results were considered significant after correction at the 0.05 level.

We further investigated the correlation between clinical scores (e.g., MDS-UPDRS-III score) and the PAC values, the associated low (phase), or high frequencies (amplitude). Therefore, the median PAC value (or low or high frequency) for the vertices belonging to one RSN (e.g., motor) in all groups (e.g., PD-OFF) was obtained for each patient. Those median values were then correlated with the clinical values. This was done for all three RSNs (motor, frontal, and visual) and PAC values, as well as the low and high frequency associated with the maximal PAC value.

## 3. Results

### 3.1. Comparison of study groups

Finally, we included the data of 23 patients, of which eight were female. The mean time since diagnosis of PD was 7.0 ± 3.9 years. Furthermore, we included 24 healthy participants, of which eight were female, in our analysis. We tested for significant differences in mean age, BDI, and mini-mental state exam (MMSE) scores between the 24 controls and 23 PD patients (alpha = 0.05).

There was no significant age difference between the groups (see Table 2; Mann–Whitney U = 344.0, *p* = 0.150; Levene’s test was significant). BDI scores were significantly lower in the HC group (Mann–Whitney U = 92.5, *p* < 0.001; Shapiro–Wilk’s test was significant). Concerning the MMSE the mean score was significantly lower in the PD group (Mann–Whitney U = 366.5, *p* = 0.036; Levene’s test and Shapiro–Wilk’s test were significant). Both results are to be expected since the cognitive decline and a higher incidence of depression is common in PD patients (see also section “4.7. Limitations”).

**TABLE 2:** Test statistics.

PD vs. HC	Test results	Normality (Shapiro–Wilk)
Age (years)	Student 1.523 (*p* = 0.135)	HC: W = 0.950, *p* = 0.271 PD: W = 0.977, *p* = 0.851
BDI-II (points)	Mann–Whitney 92.500 **(*p* < 0.001)**	HC: W = 0.865, ***p* = 0.004** PD: W = 0.863, ***p* = 0.006**
MMSE (points)	Mann–Whitney 366.500 **(*p* = 0.036)**	HC: W = 0.681, ***p* < 0.001** PD: W = 0.759, ***p* < 0.001**

Test statistics of study groups. Significant results are printed in bold letters, for non-normal distributions we used the Mann–Whitney U test, for normal distributions an independent Student’s *t*-test.

Parkinson’s disease patients received, on average, 169.0 ± 37.0 mg levodopa. MDS-UPDRS-III scores improved from 36.0 ± 11.0 points in med-OFF to 21.6 ± 9.6 points in med-ON. Of the 23 PD patients 73.9% had a good levodopa response (>30% improvement of the MDS-UPDRS-III score).

For detailed descriptive statistics, see Table 1.

### 3.2. Network comparison

We found three RSNs in all jackknife runs: the SMN, frontal, and visual RSN (see Figure 1 and also see Table 3 for the MNI coordinates of the highest coupling strength within each RSN). Comparison between conditions yielded significant differences in the coupling strength of all three RSNs, at least in the comparison of HC versus PD-OFF.

**TABLE 3:** MNI coordinates of resting-state networks hot spots.

RSN	Study group	MNI coordinates in (m)
Motor	PD-OFF	0.021/−0.027/0.056
	PD-ON	0.026/−0.037/0.052
	HC	0.015/−0.043/0.054
Visual	PD-OFF	0.034/−0.072/−0.013
	PD-ON	−0.024/−0.076/−0.007
	HC	0.023/−0.074/−0.010
Frontal	PD-OFF	−0.007/0.070/0.010
	PD-ON	−0.001/0.007/0.068
	HC	−0.019/0.037/0.040

MNI coordinates of the highest coupling strength of each resting-state network (RSN) for each study group.

#### 3.2.1. Sensorimotor network

**FIGURE 2:**
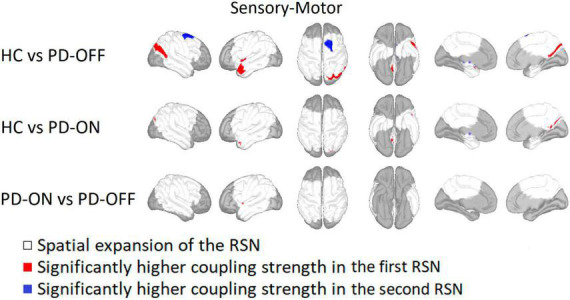
Comparison of the sensorimotor network. Sensorimotor network comparison between Parkinson’s patients with medication (PD-ON), without medication (PD-OFF), and healthy participants (HC). Areas belonging to either one and/or both recording conditions are marked in white. Red indicates a significantly higher coupling strength for the former measurement group and blue for the latter one. Significance is given at a *p*-value below 0.05 after false discovery rate correction for the number of vertices, networks, and conditions based on a two-sided Student’s *t*-test (HC vs. OFF/ON independent *t*-test, ON vs. OFF paired *t*-test). In particular, PD-OFF had a higher coupling strength in the right superior frontal gyrus than in HC, whereas the coupling strength in HC was higher in the left parietal and temporal gyrus.

For the SMN, coupling strength was significantly higher for HC on the right hemisphere in the precuneus, inferior parietal gyrus, and superior parietal gyrus compared with PD-OFF and on the left hemisphere in regions of the insula and temporal pole (see Figure 2). A higher coupling strength in PD-OFF was only present for the right superior frontal gyrus at the transition to the precentral gyrus.

Compared with PD-ON, HC showed a significantly higher coupling strength in the right precuneus and superior parietal gyrus and on the left temporal pole. The comparison between PD-OFF and PD-ON showed a significant reduction of the coupling strength in the left insula after administration of the dopaminergic medication.

#### 3.2.2. Visual network

**FIGURE 3:**
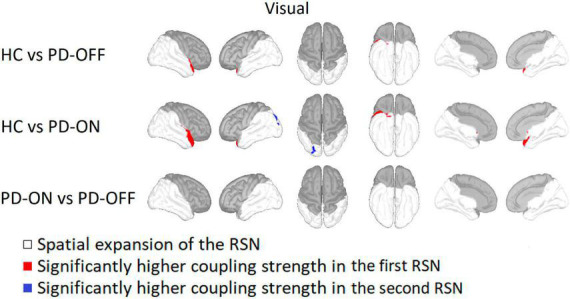
Comparison of the visual network. Visual network comparison between Parkinson’s patients with medication (PD-ON), without medication (PD-OFF), and healthy participants (HC). There were significant differences in coupling strength between all three measurement groups. Only the comparison between PD and HC showed significant differences in coupling strength. Compared to PD-OFF as well as to PD-ON, HC had a higher coupling strength at the right superior temporal gyrus. For detailed description, refer to Figure 2.

For the visual RSN, HC had a significantly higher coupling strength in the right superior temporal gyrus and right insula compared to PD-OFF and PD-ON (see Figure 3). In PD-ON, however, coupling strength in the left superior parietal and lateral occipital gyrus was higher than in HC. There were no differences between PD-OFF and PD-ON.

#### 3.2.3. Frontal network

**FIGURE 4:**
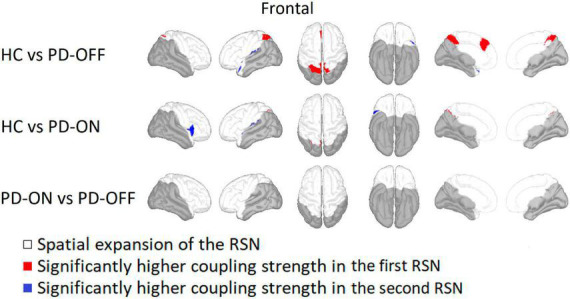
Comparison of the frontal network. Frontal network comparison between Parkinson’s patients with medication (PD-ON), without medication (PD-OFF), and healthy participants (HC). There were significant differences in coupling strength between all three measurement groups. Only the comparison between PD and HC showed significant differences in coupling strength. While PD-OFF had a lower coupling strength at the bilateral parietal gyrus than in HC, the coupling strength for PD-ON was higher than in HC, especially for the right insula. For detailed description, refer to Figure 2.

Within the frontal network the bilateral superior parietal gyrus, precuneus, and left superior frontal gyrus had a significantly higher coupling strength for HC compared to PD-OFF (see Figure 4). Sparsely, HC also showed higher coupling strength in the area of the left superior parietal gyrus compared with PD ON. At the left supramarginal and superior temporal gyrus, the coupling strength was higher for both PD-OFF and PD-ON compared to HC. For PD-ON, this was also the case for the right insula and the right pars opercularis. In contrast, there were no significant differences between PD-OFF and PD-ON.

#### 3.2.4. PAC coupling frequencies

**FIGURE 5:**
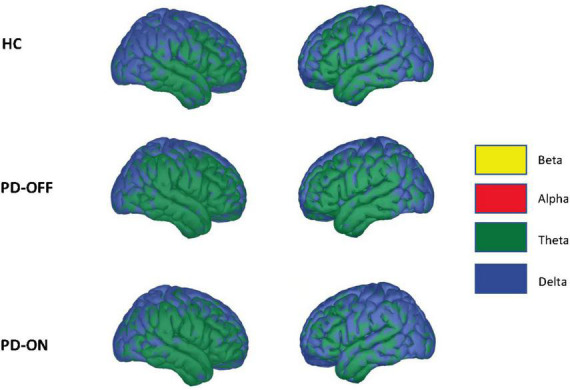
Driving low frequency component. Average low-frequency component of the phase-amplitude coupling used for network estimation across conditions. Statistically, we saw no significant differences in coupling frequencies using a two-sided independent Student’s *t*-test (false discovery rate correction, alpha = 0.05). The colors indicate the frequency spectrum at the given cortical location (delta: 2–4 Hz, theta: 4–8 Hz, alpha 8–12 Hz, beta: 12–30 Hz).

In addition to the RSNs, we also analyzed whether the frequency of the low-frequency component of the PAC differed between PD and HC groups or was altered by dopaminergic medication. There were no significant changes, neither between HC and PD-OFF nor between HC and PD-ON or PD-ON and PD-OFF groups. Overall, the low-frequencies coupling to high-gamma were in the delta and theta range (see Figure 5). We further tested for correlation between clinical values (i.e., MDS-UPDRS-III, MMSE, and BDI), and the PAC coupling frequencies, finding no significant correlations at *p* < 0.05.

## 4. Discussion

In this work, we investigated whether RSNs determined via PAC differ between HC and PD depending on the administration of dopaminergic medication. Via the chosen methods, we detected in all three groups the SMN, the visual network, and the frontal network. For all three RSNs, significant differences in coupling strength were found, especially when comparing HC and PD, but less so between PD-OFF and PD-ON.

### 4.1. Alteration in various RSNs

Due to the dominant motor symptoms in PD, differences in coupling strength were expected between the three studied groups, especially in the SMN. However, because PD is also associated with non-motor symptoms, we had as an initial hypothesis that coupling strength would be different in several functional RSNs. We were able to confirm this within the visual and frontal RSN. These findings match previous results from fMRI and also multimodal approaches, which indicated changes in different RSNs in PD (Filippi et al., 2019; Ruppert et al., 2021; Steidel et al., 2022).

In terms of PD-related impairment, there were significant differences in motor (MDS-UPDRS-III) as well as non-motor (BDI, MMSE) scores in our patient cohort. Thus, the differences in PAC-RSNs coupling strength could be electrophysiological correlates of the clinical scores. However, there was no significant correlation between the coupling strength values and the clinical scores. This may be due to used scores not being specific to a particular functional network. For example, we did not obtain scores related to visual impairments. A repeated analysis with scores functionally matching the considered RSNs could establish the link between RSN changes and scoring results.

Overall, this confirms that RSNs in PD determined via PAC are sensitive to changes in the patient’s condition, e.g., the medication state, which was already evident when comparing RSNs before and after electrode implantation (Sure et al., 2023). In the present case, however, it is striking that the changes occurred mainly between HC and PD and less between PD-OFF and PD-ON. While there was a significant change between PD and HC for all three RSNs, this was only the case for the SMN for PD patients with and without dopaminergic medication. The dopamine-related difference in coupling strength of the SMN emerged only in the area of the left insula, where sensorimotor information, besides a wide variety of other functions, are processed (Uddin et al., 2017). However, at the insula, which is located at the periphery of the SMN, the overall coupling strength is lower than in the center of the SMN. As a result, the influence of this alteration in the coupling strength on the motor system is likely to be less relevant. Therefore, a normalizing effect on RSNs by dopaminergic medication as in previous work (Schneider et al., 2020) can only be confirmed with these data for the SMN but not for the visual and frontal RSNs. The absence of this difference may be related to the different approaches used for estimating the RSN: in Schneider et al. (2020) we used envelope correlation in specific frequency bands, while here we used PAC, which incorporates both low (delta and theta) as well as high (gamma) frequency oscillatory activity. Therefore, the changes in the previous study are likely tight to a specific frequency band and the PAC might not be the best marker to indicate the changes in dopamine related changes of the RSN.

### 4.2. Sensorimotor network

In the SMN, we detected a modulation of the coupling strength, especially in comparing HC and PD-OFF. Coupling strength increased, in particular within the supplementary motor area for PD-OFF, which is in line with the already described increase of the PAC values in this area (De Hemptinne et al., 2013). However, in this case, the frequency contributing to the PAC signal was in the beta range, whereas in our case, it was in the theta/delta range. Even though the beta band is considered very important for the motor function in PD, connections to the motor function could also be established for the theta/delta band (Brauns et al., 2014; Hamel-Thibault et al., 2018; van der Cruijsen et al., 2021). Since cortical network formation in PD also occurs in the theta/delta band (Sharma et al., 2021), this work further highlights that when looking at PAC in PD, broadband oscillatory activity, i.e., not only beta, should be considered.

For HC, coupling strength was higher compared to PD-OFF within two separate regions, the left temporal and right occipital/parietal. At the same time, coupling strength was higher in the supplementary motor area in PD-OFF, suggesting PD-induced modulation of the SMN. Here, the peripheral areas seem to be less important with respect to the coupling strength, while central areas are strengthened. This fits with higher PAC in the primary motor cortex of PD patients (De Hemptinne et al., 2013) and may reflect the prominent effect of PD on the motor system.

### 4.3. Visual network

Like many other parts of the cortex, also alterations within the visual system have been described in PD. Clinically visual hallucinations are often encountered in PD patients (Barnes and David, 2001; Meppelink et al., 2009). However, visual hallucinations occur mainly in severely advanced PD and, moreover, the role of dopaminergic medication in the development of hallucinations in PD is not fully elucidated (Powell et al., 2020). Nevertheless, surprisingly, coupling strength in the superior parietal and lateral occipital gyrus – areas around the visual cortex – was only increased when comparing HC and PD-ON. For PD-OFF, coupling strength was only altered in the temporal lobe. Still this dopaminergic effect on the visual RSN could be a further indication for the central role of dopamine in the visual system, as already shown anatomically in the retina (Harnois and Di Paolo, 1990; Jackson et al., 2012) or functionally in cognitive vision (Vitay and Hamker, 2007). Furthermore, the increased coupling strength for PD-ON could also be a reflection of the saccadic latency being prolonged by dopamine (Michell et al., 2006).

We did not find significant differences in the visual network comparing PD-ON versus PD-OFF. Schneider et al. (2020) described a shift in main frequency for the visual network component, i.e., in the delta band for HC and alpha band for PD patients OFF medication. In their investigation, levodopa was able to reinstate the physiological delta rhythm in PD patients. With the PAC-based network extraction, network components are not extracted separately for each frequency band. Since visual impairment is tied to whole-brain alteration in PD for both patients with and without visual hallucinations, significant changes in PAC that are related to visual impairments may be detectable in other network components than the ones we considered in this study.

### 4.4. Frontal network

The network described in this article as a frontal network approximates the well-known DMN (Mantini et al., 2007; Brookes et al., 2011), which has been investigated in the context of several diseases, revealing altered DMN activity in various neurological diseases like Alzheimer’s disease, epilepsy, and also PD (Mohan et al., 2016). Since dopamine has a modulatory effect on the DMN in face recognition (Delaveau et al., 2010), an influence on the frontal network described by us was expected due to the spatial similarity. Although there was no difference between PD-OFF and PD-ON for the frontal network, there were partially changes of coupling strength between PD-OFF and PD-ON compared to HC, suggesting an influence of dopamine on the network. Lacking a difference in coupling strength for the frontal network between PD-OFF and PD-ON, dopaminergic medication is not likely to have a normalizing effect, as it can be assumed for the SMN. Moreover, it can be assumed that there is no general attenuation or amplification of the frontal network when comparing PD and HC but rather a spatial modulation. This is because areas close to the sulcus centralis HC had higher coupling strength than in PD, and more distant areas tended to have higher coupling strength in PD compared to HC.

### 4.5. Driving low frequencies

The choice of the megPAC approach for network determination was made because previous studies have reported that the coupling between beta phase and high gamma amplitude is pathologically modulated in PD, and thus PD-specific RSN changes could be accentuated (De Hemptinne et al., 2013; Swann et al., 2015). These alterations are, however, most likely linked to a change in the non-sinusoidal characteristics of the oscillations and not PAC itself (Cole and Voytek, 2017). Still, the change of oscillations and the change in PAC, be it due to sinusoidal or non-sinusoidal characteristics, is an indication of pathologically changed brain activity. Of note here is that we did not test in the present paper for a non-sinusoidal character of the oscillations (Cole and Voytek, 2017). The main reason is that current methods to do these kinds of tests are not computationally feasible with the large number of signals we are analyzing, i.e., 15,002 per subject. Of note, the low-frequency strongest coupling to gamma is not significantly different between the patients and HC. Those are the signals which are the basis for the extraction of the megPAC-based RSN. Thus, the estimation of the RSN is based on more or less the same low-frequency component, making it likely not the driving effect of the RSN differences we report.

In our study, the PAC low-frequency was not in the beta band but in the delta/theta band. Of note here is that Swann and colleagues did not investigate the delta frequencies, and thus the findings are not directly comparable. Especially within the motor areas, where Swann et al. (2015) and De Hemptinne et al. (2013) report most of the changes in PAC, we determined delta as the main driving low-frequency from the PAC.

In addition, network formation in the delta/theta band could be detected in non-human primates under pharmacologically induced PD (Devergnas et al., 2019), PD patients under dopaminergic medication (Sharma et al., 2021), and also other measurement groups (Babiloni et al., 2017; Harper et al., 2017; Muthukrishnan et al., 2020). Since the low-frequency component of the PAC signal was in the delta/theta band in all three conditions with no significant difference between groups, in our comparison, network formation is based mainly on delta/theta activity. An indirect influence between our determined delta/theta PAC and beta is conceivable because PAC between theta and beta has been shown for PD patients (Karimi et al., 2022).

### 4.6. Clinical applications

If levodopa effects on RSNs can reliably be derived from electrophysiological data such as with megPAC, new opportunities for clinical applications arise. Especially the objective nature of such measurements could be useful, e.g., in cases where the patients’ own judgment might be limited due to their disease. It has been shown for instance, that in up to a quarter of PD patients dyskinesias due to overdosing of levodopa are not properly recognized by the patient (Amanzio et al., 2010; Pietracupa et al., 2013).

### 4.7. Limitations

Parkinson’s disease patients had significantly higher BDI-II scores and lower MMSE scores. These differences in depression scores and cognitive abilities should be kept in mind when interpreting our findings. However, PD is also known to be associated with non-motor symptoms, which is also likely to affect functional networks.

It can be assumed that patients were in a dopaminergic OFF state because the patients were measured 12 h after the last administration of therapeutic medication for PD. However, nine patients received dopamine agonists [levodopa equivalent daily dose: 62.7 ± 112.9 mg (mean ± SD), calculated according to Schade et al. (2020)], which have a longer half-life. Therefore, it is possible that residual medication was present and influenced the OFF state so that the OFF state might not be the same for each patient. Yet, a washout period of 12 h is common practice in PD research. Furthermore, for the ON state, two patients received their regular dopaminergic medication and no fast-acting soluble levodopa. As this was the case for only two patients, this should not significantly impact the results.

Compared to Schneider et al. (2020), we could not find significant differences in the visual network after levodopa intake. This might be because we did not calculate the networks for different frequency bands separately. Thus, the changes within the visual network might only occur within a small frequency range, which might not be detectable by the PAC-based network extraction. With regard to other PAC studies on PD, our results are not completely comparable since most PAC studies in PD are investigating phase-amplitude coupling between a beta-phase and amplitude of high-gamma-oscillations, whereas the phase-driving low-frequency component in our study was in the delta to theta range.

Since subtype- and lateralization-specific changes in brain networks have been described for PD patients (Wang et al., 2016; Riederer et al., 2018), it would have been desirable to also compare the networks for different subtypes and disease lateralization. However, our sample was too small for such a comparison. Future studies with a larger sample should investigate this.

Furthermore, it would be interesting to confirm our findings within the frontal network/DMN using a task-based paradigm to assess activation/deactivation within the DMN. This would also allow for better comparability with other studies.

## 5. Conclusion

We determined significantly altered PAC RSNs in PD patients compared to HC and that dopaminergic medication has a normalizing effect for the SMN. Therefore, in extension to Florin and Baillet (2015) and Sure et al. (2023), PAC-RSN are present in both healthy and patient groups and RSN of different groups can be compared. Since Schneider et al. (2020) indicated a normalization effect for RSN with other methods, our results suggest that this effect is independent of the method used for RSN estimation. Nevertheless, while there are changes in coupling strength, the low-frequency component driving the PAC remains unchanged between HC and PD patients both ON and OFF medication. Our results indicate a role of PAC RSNs both in the pathogenesis of PD beyond the sensorimotor system as well as the effect of levodopa on motor symptoms. Our data-driven, PAC-based approach is thus in line with previous work based on fMRI and EEG, highlighting the potential of MEG-driven paradigms for investigating electrophysiological changes in PD.

## Data availability statement

The datasets presented in this article are not readily available because of privacy law, data are available upon reasonable personal request. Enquiries can be sent to the corresponding author. Requests to access the datasets should be directed to EF, esther.florin@hhu.de.

## Ethics statement

The studies involving human participants were reviewed and approved by Ethics Committee of the Medical Faculty, Heinrich Heine University Düsseldorf. The patients/participants provided their written informed consent to participate in this study. Written informed consent was obtained from the individual(s) for the publication of any potentially identifiable images or data included in this article.

## Author contributions

SM: software, validation, formal analysis, investigation, data curation, writing—original draft, and visualization. MS: software, validation, formal analysis, investigation, and writing—review and editing. AS: methodology, resources, and writing—review and editing. EF: conceptualization, methodology, validation, formal analysis, investigation, data curation, writing—review and editing, supervision, project administration, and funding acquisition. All authors contributed to the article and approved the submitted version.

## Footnotes

1
http://neuroimage.usc.edu/brainstorm
